# Nursing in Workhouses

**Published:** 1897-05-29

**Authors:** 


### EDITOR'S LETTER-BOX.

[Our Correspondents are reminded that prolixity is a great bar to publication, and that brevity of style and conciseness of statement greatly facilitate early insertion.]

### NURSING IN WORKHOUSES.

Dr. H. W. Webster, Medical Superintendent of the St. George's Union Infirmary, Fulham Road, writes: My attention has been called to an article, entitled "Nursing in Workhouses," which appeared in The Hospital of May 8th, 1897. A statement is there made—on what authority I know not—that in the St. George's Infirmary there is only one trained nurse to teach twenty-five probationers. As this statement is quite untrue, and calculated to do harm to the prospects of those nurses now in the infirmary, and also to diminish the number of applications from desirable candidates, I must ask you to kindly publish the following correction: There are in the infirmary at the present time seventeen trained and certificated nurses. There are six more who have attended all the necessary lectures and demonstrations, and who have passed their examinations in a satisfactory manner. (The examinations are conducted by an independent medical examiner appointed by the board.) Certificates are now being prepared for these nurses. Since the beginning of December last eleven trained and certificated nurses have left the infirmary to take other appointments. I may add that the last matron, Miss Griffiths, was a hospital-trained lady of very wide experience, and that she devoted herself with great energy to the teaching of the probationers. The present matron, Miss Hampson, a lady of undoubted ability, trained at St. Thomas's Hospital, will, I am sure, make the teaching at St. George's Infirmary equal to that given at any metropolitan infirmary. The new home which the guardians have decided to build, with separate rooms for seventy nurses, and all modern requirements for their comfort and instruction, will place their infirmary in a very advantageous position among similar institutions.

⁂ If Dr. Webster had read the article he criticises with the slightest care he could not have remained ignorant of the authority on which our statement was made, which, as we stated, was a Parliamentary return made to the House of Commons. If there is any error in this return, which referred to the year 1896, someone connected with the infirmary must have made the blunder complained of. The blame of this, however, must lie on their shoulders, not on ours. We took our figures from a public official document.—Ed. *T. H.*

